# Maternal Vitamin D Status and Gestational Weight Gain as Correlates of Neonatal Bone Mass in Healthy Term Breastfed Young Infants from Montreal, Canada

**DOI:** 10.3390/nu13124189

**Published:** 2021-11-23

**Authors:** Nathalie Gharibeh, Maryam Razaghi, Catherine A. Vanstone, ShuQin Wei, Dayre McNally, Frank Rauch, Glenville Jones, Martin Kaufmann, Hope A. Weiler

**Affiliations:** 1 School of Human Nutrition, McGill University, Ste-Anne-de-Bellevue, QC H9X 3V9, Canada; nathalie.gharibeh@mail.mcgill.ca (N.G.); maryam.razaghi@mail.mcgill.ca (M.R.); catherine.vanstone@muhc.mcgill.ca (C.A.V.); 2 Institut National de santé Publique du Québec, Montréal, QC G1V 5B3, Canada; shu-qin.wei@inspq.qc.ca; 3 Department of Pediatrics, Children’s Hospital of Eastern Ontario, University of Ottawa, Ottawa, ON K1H 8L1, Canada; dmcnally@cheo.on.ca; 4 Shriners Hospital for Children, Montréal, QC H4A 0A9, Canada; frauch@shriners.mcgill.ca; 5 Department of Biomedical and Molecular Sciences, Queen’s University, Kingston, ON K7L 3N6, Canada; gj1@queensu.ca (G.J.); martin.kaufmann@queensu.ca (M.K.); 6 Nutrition Research Division, Bureau of Nutritional Sciences, Health Products and Food Branch, Health Canada, Ottawa, ON K1A 0K9, Canada

**Keywords:** neonate, mother, vitamin D, 25-hydroxyvitamin D, gestational weight gain, bone mineral content, bone mineral density

## Abstract

The implications of maternal gestational weight gain (GWG) and vitamin D status to neonatal bone health are unclear. We tested whether maternal 25-hydroxyvitamin D (25(OH)D) and GWG relate to neonatal bone mineral content (BMC) and bone mineral density (BMD). Healthy term appropriate for gestational age breastfed neonates (*n* = 142) and their mothers were recruited 24–36 h after delivery and followed at 1.0 ± 0.5 month. At birth, obstetric data were collected and newborn serum 25(OH)D was measured. At 1 month, neonatal whole-body (WB) BMC, WB BMC relative to body weight (WB BMC/kg), lumbar spine BMC and BMD, maternal and neonatal 25(OH)D concentrations, and anthropometry were measured. Infant BMC and BMD between maternal 25(OH)D (<50, ≥50 nmol/L) and GWG (insufficient, adequate, and excessive) categories were compared. Maternal 25(OH)D was not related to infant whole-body BMC, BMC/kg, lumbar spine BMC, and BMD. Infants in the excessive maternal GWG category had greater (*p* = 0.0003) whole-body BMC and BMC/kg and lumbar spine BMC and BMD than inadequate GWG, and greater (*p* = 0.0063) whole-body BMC/kg and lumbar spine BMC and BMD than adequate GWG. These results suggest that maternal GWG, but not vitamin D status, modestly relates to bone mass in neonates.

## 1. Introduction

A significant amount of variance in peak bone mass remains unexplained by genetic and lifestyle factors [1,2], and is postulated to be attributed to skeletal programming in utero [3]. This is exemplified in the Avon Longitudinal Study of Parents and Children in which maternal exposure to ultraviolet B (UVB) radiation and folate intake during pregnancy were both positively related to childhood (9 years of age) bone mineral content (BMC) and bone mineral density (BMD) [4]. Other maternal factors related to an offspring’s bone outcomes include maternal smoking [5] and alcohol consumption [6] that are negatively related to bone outcomes, whereas the relation of maternal vitamin D supplementation [7,8,9], vitamin D status [10,11,12,13], pre-pregnancy body mass index (BMI), and gestational weight gain (GWG) [14] to infant bone outcomes is equivocal.

Indicators of nutritional status of the mother, including pre-gravid BMI and GWG, have been emphasized in the US Institute of Medicine (IOM) guidelines for a healthy pregnancy [15]. In order to optimize the health of infants and their chances of being born appropriate for gestational age (AGA), GWG targets have been set by the IOM according to pre-gravid BMI. Accordingly, GWG is described as being insufficient, adequate, and excessive. Nonetheless, the impact of maternal pre-gravid BMI and GWG on an offspring’s growth parameters including skeletal growth remains inconclusive. One study from the UK suggests that the positive associations between pre-gravid BMI and child BMC and BMD at 9 years of age are possibly due to genetic and environmental influences throughout childhood rather than intrauterine exposures [16]. Another study found that GWG positively associates with BMC of the offspring at 7 years of age in mothers with a pre-gravid BMI < 25 kg/m^2^, but not in mothers with pre-gravid BMI ≥ 25 kg/m^2^ [14]. As such, the relation of pre-gravid BMI and excessive GWG to offspring bone outcomes is unclear. Excessive GWG essentially translates into excess adiposity; which is well known to be associated with higher BMC and BMD in children [17] and adults [18], but may limit the availability of vitamin D and potentially bone health of the offspring. Sequestration of vitamin D in adipose tissue [19] could result in lower circulating serum 25-hydroxyvitamin D (25(OH)D) concentrations in the mother and potentially a decreased transfer of 25(OH)D from the mother to the fetus.

Vitamin D stores of newborn infants are a function of maternal-fetal transfer of 25(OH)D during gestation [20] and are usually lower in the infant compared to the mother [11,21,22]. While some epidemiological studies report a positive relationship between maternal 25(OH)D status and bone outcomes in infancy [10,12,23,24], childhood [25], and adulthood [26], others do not [27,28,29,30,31]. In a cohort of 50 newborn infants and their mothers in Winnipeg, Canada, neonates with insufficient vitamin D status at birth have lower whole-body bone mineral content (BMC) relative to weight (BMC/kg) [11], suggesting that maternal vitamin D status may affect offspring bone health.

To our knowledge, previous studies have not explored how both maternal vitamin D status and GWG relate to neonatal bone outcomes. The objective of this study was to determine whether neonatal bone mass in healthy term, breastfed infants differs according to maternal vitamin D status and GWG. We hypothesized that sufficient maternal vitamin D status and healthy pregnancy weight gain associate with greater BMC/kg compared to insufficient maternal vitamin D status and insufficient or excessive GWG.

## 2. Methods

### 2.1. Sample and Study Design

This study was based on the newborn and 1 month postpartum data from a double-blind randomized controlled trial on vitamin D supplementation in infancy registered with clinicaltrials.gov (NCT02563015). Mother–infant dyads were recruited within 24–36 hours after delivery at the Lakeshore General Hospital, Montreal, Canada (March 2016–March 2019). Inclusion criteria were healthy, term, AGA singleton infants of a healthy pregnancy and whose mothers had an intent to breastfeed for at least 3 months. Exclusion criteria were maternal diseases (diabetes (any type), pre-eclampsia, celiac disease, and inflammatory bowel disease), smoking during pregnancy, and the use of prescription medication(s) that alter vitamin D and/or bone metabolism and/or fetal growth. At the hospital, neonatal capillary blood was sampled for subsequent measurement of serum 25(OH)D concentrations. The sample collection occurred during clinical screening for inborn errors of metabolism (e.g., phenylketonuria) in order to minimize the frequency of blood sampling.

At the hospital, data on obstetric history, anthropometric measurements at birth, and demographics were collected. The bone health of the neonate, nutritional intake of the mother during gestation, serum 25(OH)D concentrations, and anthropometry of the neonate and mother were assessed at 1.0 ± 0.5 months postpartum at the Mary Emily Clinical Nutrition Research Unit of McGill University, Montreal.

### 2.2. Obstetric History, Anthropometric, and Demographic Data

Obstetric history, including mother’s age, height, weight (prior to conception and at delivery), gravida, and pre-gravid BMI, were collected from the medical records and confirmed by a maternal postpartum survey. GWG was calculated as: weight at delivery minus weight prior to conception (kg), and the categories (inadequate, adequate, and excessive) were defined according to the IOM’s classification [15]. Based on pre-gravid BMI < 18.5 kg/m^2^, the recommended adequate GWG is 12.7 to 18.2 kg; if BMI was between 18.5 to 24.9 kg/m^2^, adequate GWG is 11.4 to 15.9 kg; if BMI was between 25.0 and 29.9 kg/m^2^, adequate GWG is 6.8 to 11.4 kg; and if BMI was 30 kg/m^2^, adequate GWG is defined as 5 to 9.1 kg. Values for GWG below the lower range for each BMI category are considered inadequate; and values for GWG above the upper range are considered excessive (Supplementary Figure S1). Mothers were also grouped into categories according to their pre-gravid BMI: <25 kg/m^2^ and ≥25 kg/m^2^.

Data on date of birth, sex, gestational age at birth, and birth weight of the infants were obtained from the medical charts. Birth length and head circumference data were not used as the measurements were not standardized and thus were not reliable. At the postnatal study visit, infant nude weight (dynamic scale, Mettler-Toledo Inc., Columbus, OH, USA) was measured to the nearest gram and length (infantometer, O’Learly Length Boards, Ellard Instrumentation Ltd.) and head circumference (non-stretchable tape, Perspective Enterprises) were measured to the nearest 0.1 cm. Corresponding z-scores (World Health Organization AnthroPlus Software 2009, Geneva, Switzerland) were calculated. The mother’s weight (balance beam scale, Detecto; Webb) and height (stadiometer, Seca Medical Scales and Measuring Systems) were measured to the nearest 0.1 kg and 0.1 cm, respectively.

Demographic data were collected using a postpartum survey designed according to Statistics Canada descriptors [32,33] and for the purpose of better understanding the health needs of different populations. Data on the mother’s self-reported population group (white, all other: South Asian, Chinese, Black, Filipino, Latin American, Arab, Southeast Asian, West Asian, Korean, Japanese, and other), education (high school, college, or university), in addition to annual family income (<70,000 CAD, ≥70,000 CAD, or not reported) were obtained.

### 2.3. Dietary Intake, Multivitamin Use, and Physical Activity

Nutritional intake during all three trimesters of pregnancy combined was assessed using a validated semi-quantitative food frequency questionnaire [34] given the potential relation of maternal intake and GWG during pregnancy to offspring bone outcomes [35,36]. The data was analyzed using the Canadian Nutrient File. Data on intake of total energy, protein, carbohydrates, fat as well as calcium, vitamin D, magnesium, and phosphorus from both food and supplements were explored as continuous variables. Daily intake of these nutrients (g or mg per 1000 kcal) normalized for energy intake was also tested. Data on use of multivitamins and physical activity during pregnancy (Yes/No) were collected using the maternal postpartum survey.

### 2.4. Serum 25(OH)D Concentration

Infant capillary blood samples (0.5 mL) were collected (at birth and at 1.0 ± 0.5 months of age) by heel lance. Maternal venous blood (5 mL) was collected at the postnatal visit. The samples were centrifuged (4000× *g*, 6 °C) for 20 min and serum was collected and stored at −80 °C until 25(OH)D concentrations were measured. The serum 25(OH)D concentrations were measured using an automated chemiluminescent immunoassay (Liaison, Diasorin Inc.) and standardized to the National Institute of Standards and Technology (NIST) reference measurements [37] using Deming regression (standardized concentration (nmol/L) = 0.9634 measured concentration (nmol/L) + 3.122). The serum 25(OH)D concentrations were classified as sufficient (≥50 nmol/L) or insufficient (<50 nmol/L) as per the IOM’s recommendations [38]. The laboratory participated in the Vitamin D External Quality Assessment Scheme and obtained a certificate of proficiency. Using the NIST Standard Reference Materials 972a Level 1–4 quality control samples, the accuracy was 97.4%. The precision was measured using both NIST972a and internal laboratory controls; with inter-assay % CV <10% for both. The total serum 25(OH)D from a subgroup of mother–infant dyads (*n* = 83) measured using chemiluminescent immunoassay agreed well (mean difference = −0.75 nmol/L) with concentrations obtained using liquid chromatography tandem mass spectrometry from a laboratory (Queen’s University, Kingston, Canada) certified by the DEQAS Certification Program.

### 2.5. Bone Outcomes

Whole-body BMC, lumbar spine (1–4) BMC, and BMD were measured using dual-energy X-ray absorptiometry (DXA, fan beam, APEX 13.3:3, Hologic 4500A Discovery Series, Hologic Inc., Bedford, MA, USA) in array mode as recommended [39]. Whole-body and lumbar spine scans were obtained using infant whole-body mode and anterior–posterior (AP) spine mode, respectively. The regions of interest, including head and lumbar spine (1–4), were defined using manual bone edge detection. The precision was measured using a Hologic spine phantom (No. 14774) and % CV for each of BMC, BMD, and bone area were <1%. Neonates were dressed in light gowns and diapers, covered with light blankets, and rocked to sleep prior to scan acquisition.

### 2.6. Ethical Approval

This study was reviewed and approved by the St. Mary’s Hospital Research Ethics Committee which oversees ethics at the Lakeshore General Hospital (SMHC 15–34). The study was also reviewed and approved by the Health Canada Research Ethics Board (REB 2019-033H) and the Privacy Management Division (HC-PR-2019-000024). The study was conducted in accordance with the Declaration of Helsinki. All of the study materials were available in English and French, the two official languages in Canada. Written informed consent was provided by the families at the hospital prior to participation in the newborn screening and at McGill University prior to enrolment in the vitamin D trial.

## 3. Statistical Analysis

The sample size was calculated based on differences in neonatal whole-body BMC using 5% significance level (α = 0.05), power of 95%, an effect size of 0.2, and considering a total of 9 variables included as fixed effects in the models tested: infant: sex, age, and length-for-age z-scores (LAZ) at the 1 month postpartum visit; maternal: 25(OH)D categories, pre-pregnancy BMI categories, GWG categories, education, self-reported population group, and annual family income. The minimum sample size required was 127. To account for the risk of motion artifacts, given that keeping infants still during scans is often challenging, an additional 10% was considered and a total of 140 mother–infant dyads was set as the target sample size.

Data are reported as mean +/− SD or mean (95% CI) or median (IQR) for continuous variables or percentages for categorical variables. In addition to the mean +/− SD, the full data range (minimum-maximum) was also provided for the 25(OH)D concentrations of infants and mothers (nmol/L) in different 25(OH)D categories and for GWG (kg) in each GWG category. Infant vitamin D status reflects maternal fetal transfer and vitamin D supplementation, and we therefore tested the relation between maternal and neonatal vitamin D status at birth and 1 month using a mixed linear model (PROC MIXED). The differences between maternal 25(OH)D categories and GWG categories in whole-body BMC (g) and BMC per body weight (BMC/kg; g/kg), lumbar spine BMC (g), and BMD (g/cm^2^) were tested using a mixed linear model. The variables tested as fixed effects were considered based on previous research, including infant sex [40,41], age [42], and length-for-age z-scores (LAZ) [43]; maternal characteristics: 25(OH)D categories [11,24], pre-pregnancy BMI categories [14], GWG categories [38], gravida [44], and age [45]; and sociodemographic characteristics [46,47,48]: maternal education and self-reported population group, and annual family income. Given that GWG may be an effect modifier of the maternal vitamin D status–infant bone outcomes association [19], both factors were tested in the same model. The regression coefficients (95% CI) for these variables are reported. The model fit was evaluated using the Bayesian information criterion (BIC) and R-squared values. The testing of normality of residuals was done using Shapiro–Wilk test. Tukey–Kramer tests were used for post hoc comparisons with adjustment for multiple comparisons (i.e., three GWG categories) and Chi square or Fisher’s exact test for categorical variables. While the recommended ranges for maternal serum 25(OH)D, pre-gravid BMI, and GWG formed the main analysis, these variables were also tested as continuous data. The means of the different macro and micronutrient intakes consumed by the mothers during gestation were compared between the GWG categories in a subgroup analysis using a mixed linear model with the data expressed as absolute values and normalized for energy. All of the statistical analyses were conducted using SAS University Edition (SAS Institute Inc., Cary, N.C.) and the statistical significance was set at *p* < 0.05 after adjustment for multiple comparisons.

## 4. Results

**Figure 1:**
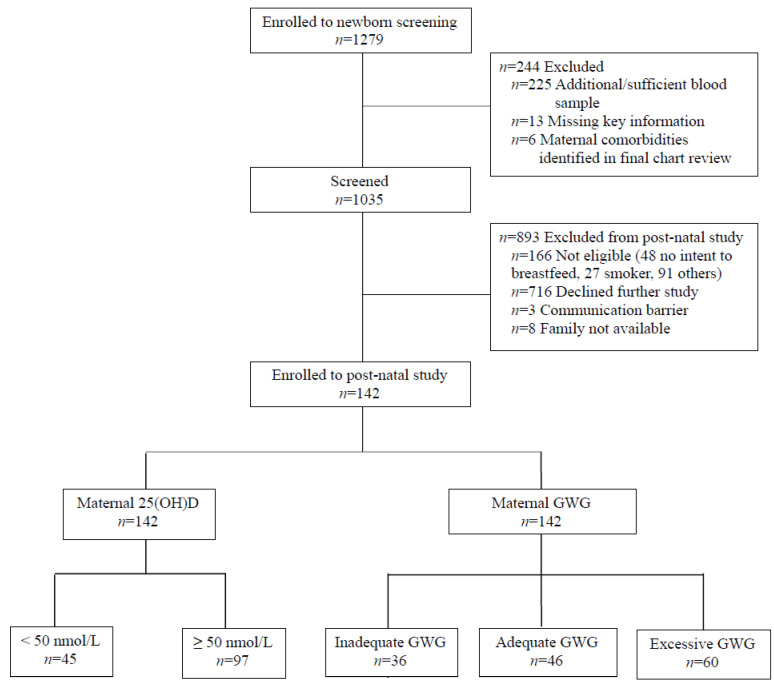
Participant flow diagram. Participant flow diagram illustrating the number of mother–infant dyads assessed for eligibility 24–36 h after delivery, enrolled in newborn screening, screened, and enrolled in postnatal study with distribution according to maternal vitamin D status (insufficient vs. sufficient) and GWG categories (inadequate, adequate, and excessive). Abbreviations: 25(OH)D: 25-hydroxyvitamin D, and GWG: gestational weight gain.

Out of 1035 infants tested for newborn vitamin D status, a total of 142 mother–infant dyads participated in the postnatal assessment (Figure 1); the comprehensive participant flow diagram is published elsewhere [49]. The infants were born at 39.2 ± 1.1 weeks of gestation to mothers 32.2 ± 4.4 years of age, with 58.5% of the infants born between April 1 and October 31. The maternal and infant characteristics were not different among groups of maternal 25(OH)D concentrations except for maternal self-reported population group and education (Table 1). Overall, 92.2% (*n* = 131) of the mothers reported they took multivitamins, and 49.3% (*n* = 70) exercised during pregnancy. In terms of infant characteristics as per GWG categories, at birth, infants of mothers with inadequate GWG had lower weight-for-age z-scores (WAZ) compared to those in the adequate and excessive GWG categories. Differences between inadequate and excessive GWG categories in WAZ were carried over to the neonatal phase. In addition, at 1 month, infants born to mothers with excessive GWG had higher LAZ compared to the mothers with inadequate GWG. As for maternal characteristics, the proportions of mothers with pre-pregnancy BMI <25 and ≥25 kg/m^2^ varied among groups of GWG (Table 1). No differences in sociodemographic characteristics were noted between the GWG categories. Intake of energy, protein, fat, carbohydrates, vitamin D, calcium, phosphorus, and magnesium did not vary according to the GWG categories (Supplementary Table S1). Similarly, when normalized for energy intake (per 1000 kcal), intake of these macronutrients and micronutrients did not vary according to the GWG categories (*p* > 0.69).

**Figure 2:**
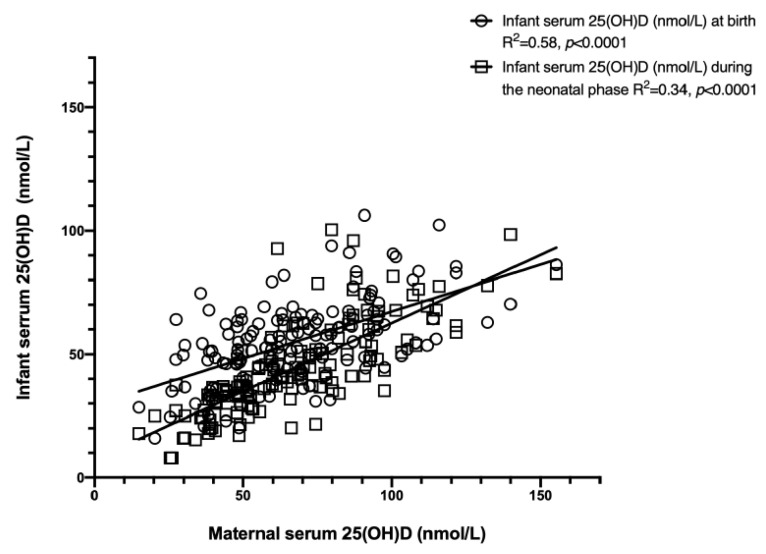
Maternal 25(OH)D concentration as a correlate of vitamin D status of neonates at birth and 1 month of age. Linear regression of infant birth (7.9–100.4 nmol/L, *n* = 142) and neonatal (15.9–106.2 nmol/L, *n* = 142) serum 25(OH)D concentrations on maternal (15.0–155.3 nmol/L, *n* = 142) serum 25(OH)D concentrations. The data were analyzed using a mixed linear model (*p* < 0.05). Abbreviations: 25(OH)D: 25-hydroxyvitamin D.

Maternal serum 25(OH)D concentrations were associated with infant serum 25(OH)D concentrations at birth and at 1 month postpartum (Figure 2). Serum 25(OH)D concentrations of the mothers were 39.9 ± 8.6 nmol/L (range: 15.0–49.9 nmol/L) and 80.0 ± 21.4 nmol/L (range: 51.2–155.3 nmol/L) in the vitamin D insufficient and vitamin D sufficient groups, respectively. In the vitamin D insufficient group, infant 25(OH)D concentrations at birth and at 1 month of age were 28.0 ± 9.4 nmol/L and 44.2 ± 14.5 nmol/L. In the vitamin D sufficient group, infant 25(OH)D concentrations at birth and at 1 month of age were 52.1 ± 17.4 nmol/L and 59.8 ± 16.0 nmol/L.

At birth, amongst infants born to mothers with 25(OH)D <50 nmol/L, 53.3% and 97.8% had 25(OH)D concentrations <30 nmol/L (range: 7.9–29.6 nmol/L) and <50 nmol/L (range: 7.9–48.3 nmol/L), respectively. At birth, 46.4% of infants born to mothers with 25(OH)D ≥50 nmol/L had 25(OH)D concentrations ≥50 nmol/L (range: 50.0–100.4 nmol/L). At the postnatal visit, 20.0% and 66.7% of infants born to mothers with 25(OH)D <50 nmol/L had 25(OH)D concentrations <30 nmol/L (range: 15.9–29.9 nmol/L) and <50 nmol/L (range: 15.9–49.8 nmol/L), respectively. The majority (73.2%) of infants born to mothers with 25(OH)D ≥50 nmol/L had 25(OH)D concentrations ≥50 nmol/L (51.2–106.2 nmol/L) at the postnatal visit. Maternal GWG was 7.1 ± 3.4 (0–11.7), 12.6 ± 2.6 (7.2–18.2), and 18.3 ± 4.1 (9.2–29.1) kg in the insufficient, adequate, and excessive GWG categories, respectively.

**Figure 3:**
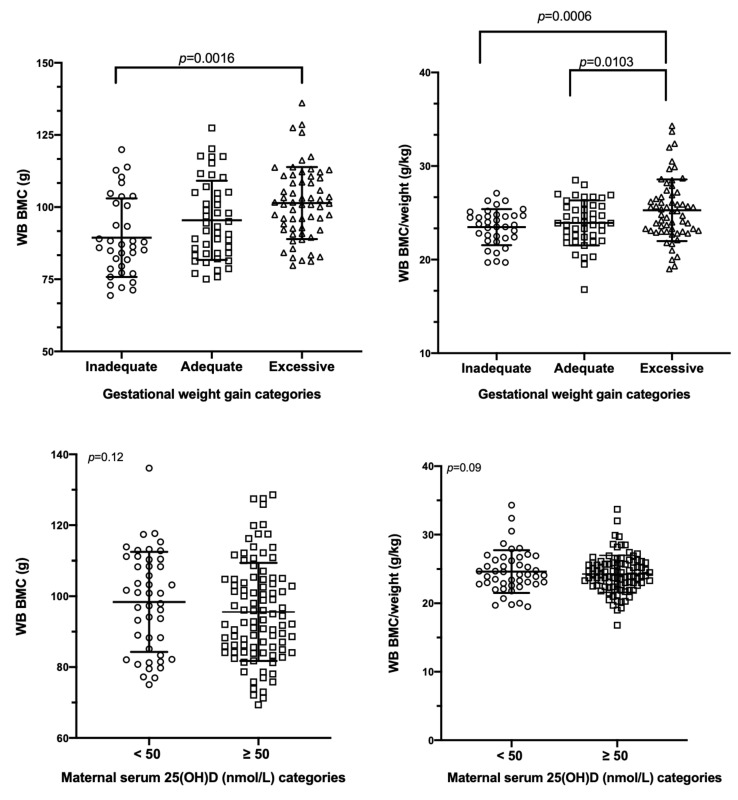
Whole-body bone mass in the neonatal period according to gestational weight gain categories and maternal 25(OH)D status. Data are reported as mean ± SD and were analyzed using a mixed linear model accounting for the fixed effects of infant’s sex, age, and LAZ score and mother’s 25(OH)D categories, pre-pregnancy BMI categories, gestational weight gain categories, education, self-reported population group as well as family income (*p* < 0.05, post hoc adjustment). Abbreviations: 25(OH)D: 25-hydroxyvitamin D, WB BMC: whole-body bone mineral content, WB BMC/kg: whole-body bone mineral content per kilogram body weight.

**Figure 4:**
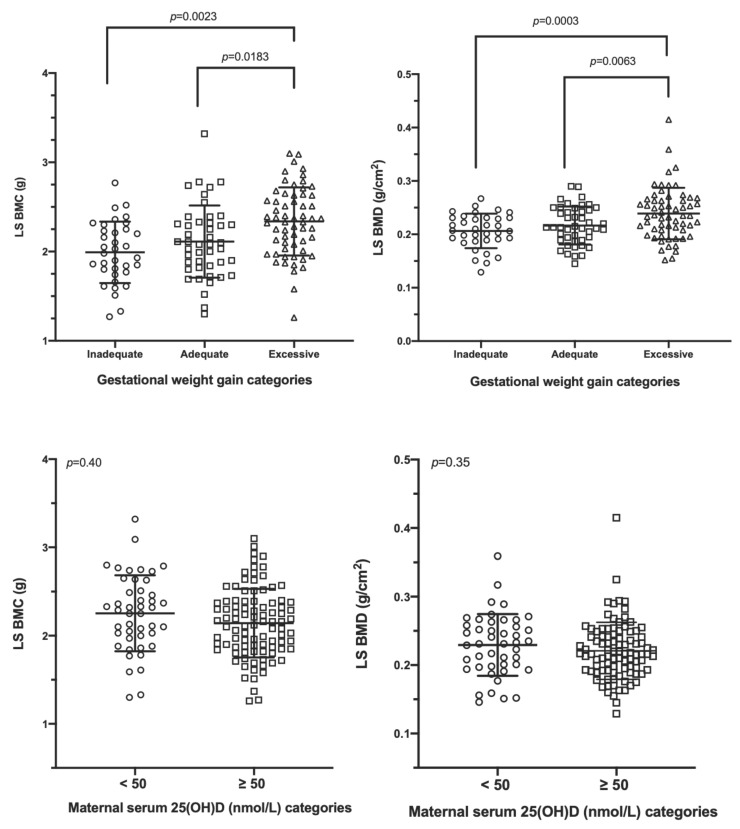
Lumbar spine bone mass in the neonatal period according to gestational weight gain categories and maternal 25(OH)D status. Data are reported as mean ± SD and were analyzed using mixed linear model accounting for the fixed effects of the infant’s sex, age, and LAZ score and the mother’s 25(OH)D categories, pre-pregnancy BMI categories, gestational weight gain categories, education, self-reported population group, as well as family income (*p* < 0.05, post hoc adjustment). Abbreviations: 25(OH)D: 25-hydroxyvitamin D, LS BMC: lumbar spine bone mineral content, and LS BMD: lumbar spine bone mineral density.

Using a mixed linear model analysis, GWG but not maternal 25(OH)D was related to neonatal whole-body BMC and BMC/kg (Figure 3) and lumbar spine BMC and BMD (Figure 4). Infants of mothers with excessive GWG had greater whole-body BMC (101.40 ± 12.47 g), whole-body BMC/kg (25.27 ± 3.29 g/kg), lumbar spine BMC (2.34 ± 0.38 g), and lumbar spine BMD (0.239 ± 0.048 g/cm^2^) than those with inadequate GWG (whole-body BMC: 89.41 ± 13.62 g; whole-body BMC/kg: 23.47 ± 1.92 g/kg; lumbar spine BMC: 1.99 ± 0.34 g; lumbar spine BMD: 0.206 ± 0.032 g/cm^2^; all *p* < 0.01). Additionally, infants of mothers with excessive GWG had greater whole-body BMC/kg, lumbar spine BMC, and lumbar spine BMD than those with adequate GWG (whole-body BMC/kg: 23.93 ± 2.41 g/kg, lumbar spine BMC: 2.11 ± 0.40 g, and lumbar spine BMD: 0.217 ± 0.036 g/cm^2^) (Figure 3 and Figure 4). Other correlates of these bone outcomes included infant sex, age, LAZ, and family income (Supplementary Table S2); in the subgroup analysis, maternal dietary intakes did not relate to any of the bone outcomes. Using adequate GWG as a referent, regression coefficients for inadequate GWG are listed in Table S2. Regression coefficients for excessive GWG with adequate GWG as the referent are: whole-body BMC: 4.32 g (95%CI: −0.08 to 8.72), whole-body BMC/kg: 1.52 g/kg (95%CI: 0.50 to 2.53), lumbar spine BMC: 0.20 g (95%CI: 0.06 to 0.34), and lumbar spine BMD: 0.023 g/cm^2^ (95%CI: 0.008 to 0.038). All of the traits had normally distributed residuals except for LS BMD. A log_10_ transformation of this variable rendered the residuals normally distributed but did not change any of the results and conclusions. Thus, for simplicity and consistency with the other traits, the untransformed data for this variable were reported. For all of these models, use of continuous data for maternal vitamin D status, pre-gravid BMI, and pregnancy weight gain results in the same interpretation (Supplementary Table S3).

## 5. Discussion

This study was undertaken to test whether maternal GWG and vitamin D status relate to the bone mass of the newborn using internationally accepted guidelines for classification of GWG, vitamin D status, and DXA scanning of infants [15,38,50]. We observed that excessive GWG related to greater BMC and BMD in healthy, term, AGA infants. These differences remained after adjustment for infant weight, suggesting that these differences are not driven by body size alone. In contrast, maternal vitamin D status did not relate to infant bone mass, only to neonatal 25(OH)D concentrations from birth to ~1 month of age. To our knowledge, no similar studies have been reported in the literature, which makes this study unique and provides insight on novel correlates for bone health in infancy that trace back to maternal exposures both preconception and during the gestational phase; an understudied area of research.

Our results show that infant whole-body BMC did not differ between inadequate vs. adequate GWG categories. This is reassuring as infants born to mothers who were not able to meet the minimum GWG recommended for their pre-gravid BMI do not seem to experience adverse bone mass outcomes at this early point in life. On the other end of the spectrum of GWG, differences between excessive and inadequate GWG categories were in part driven by body size, given the respective differences in WAZ and LAZ. As for the lumbar spine site, while differences were reported between the different GWG categories, the clinical significance of this measurement remains to be defined. The differences observed in our study in the patterns between the whole-body and the regional lumbar spine scan could be attributed to the fact that the whole-body measurement is more a function of body size versus the lumbar spine site. Indeed, after adjusting for the weight of the infant, excessive GWG was associated with greater neonatal whole-body BMC/kg compared with inadequate and adequate GWG groups. Moreover, in our study, the association of appropriateness of GWG to bone outcomes was irrespective of pre-gravid BMI and vitamin D status, highlighting the importance of the mother’s weight gain during pregnancy regardless of her bodily stores. In a study by Monjardino et al. [14], the association between GWG and bone outcomes varied according to the early pregnancy BMI categories. They [14] noted a positive association between whole-body less head BMC and GWG in women with early pregnancy BMI <25 kg/m^2^, but not in women with BMI ≥25 kg/m^2^. Additionally, they reported that in both BMI categories, weight gain in amounts exceeding the IOM’s recommendation was not shown to present any advantages for childhood bone mass. The study by Monjardino et al. may have included preterm infants with smaller size at birth as the inclusion criteria specified 24 weeks of gestational age as the cut-off for recruitment. In addition, mothers were not necessarily healthy as some had gestational diabetes and others were smokers. In contrast, our study was restricted to term AGA infants born to healthy mothers. Nonetheless, overall, both studies show modest incremental increases in BMC and BMD according to GWG categories. Interpretation of these findings should be cautious given the negative implications of excessive GWG on overall pregnancy outcomes [51].

Infant 25(OH)D concentration at birth and during the neonatal period are related to maternal vitamin D status [21,22] in addition to supplementation of the neonate [52]. The influence of maternal–fetal transfer of vitamin D becomes weaker with time, i.e., 1 month versus at birth. Counterintuitive to the role of vitamin D in calcium homeostasis, maternal 25(OH)D was not found to be a correlate of newborn bone outcomes. A study by Dror et al. (2012) [53] reported similar findings while several other studies reported a positive relation [10,11,23,24] between maternal 25(OH)D concentration and fetal, neonatal, or childhood bone outcomes. This could be explained as a function of vitamin D metabolism and exposures prenatally compared to postnatally. During gestation, intestinal calcium absorption of the mother is dependent on the active form of vitamin D or calcitriol [54], which is supported as long as 25(OH)D concentrations are not severely deficient [51], and the case for all of the mothers in our sample. The transfer of minerals, including calcium, from the mother to the fetus is also a function of other hormones, including parathyroid hormone-related protein and parathyroid hormone [55], in addition to placental and umbilical cord regulations [56]. The intrauterine metabolism of calcium contrasts that of the newborn. At birth, calcium absorption mainly occurs through a passive intestinal process that becomes more vitamin D-dependent near weaning [55]. Thus, maternal vitamin D status can have implications for offspring bone outcomes, but perhaps adverse outcomes are more evident if maternal concentrations are below the threshold of deficiency. The studies by Viljakainen et al. [10,24] and by Weiler et al. [11] were of a full spectrum of vitamin D status from deficient up to sufficient, but with greater proportions of mothers who were vitamin D insufficient (46.0–60.4%) compared to our study (31.7%). In our study, only a small proportion of mothers were vitamin D deficient (4.9%) and 92.2% of the mothers took multivitamins during pregnancy; a proportion comparable to the Canadian population (89.7%) [57]. Thus, from a clinical perspective, strategies to assure sufficient maternal vitamin D status are reinforced by this work.

This study presents with advantages and certain limitations. In addition to the unique findings reported in this study, one of its strengths is the standardization of 25(OH)D concentrations to the NIST reference measurements [37] and assessment of infant bone mass according to the ISCD [50].This study shows that the chemiluminescence immunoassay (CLIA) is suitable for measurement of total 25(OH)D in newborn serum from capillary samples, which is important in view of the recent findings on overestimation of 25(OH)D measured by CLIA in cord blood samples [58]. Some of the limitations of this study include the cross-sectional design and that maternal serum 25(OH)D concentrations were not measured during gestation or at delivery. Since fetal bone mineral accretion occurs mainly in the third trimester of gestation, we were not able to test whether maternal vitamin D status in pregnancy relates to neonatal bone mass. In addition, GWG was calculated for term deliveries and uncomplicated pregnancies as the difference between weight at delivery and pre-conception weight. This calculation overlooks the variability within trimesters as well as in late gestation, the length of which varied between 37.1 and 41.9 weeks in our study. While the IOM provides recommendations for total GWG and weekly weight gain, the methods of calculation of GWG have not been standardized [59]. The latter calls to action for a better comparison of GWG related findings across studies and for a more accurate assessment of GWG in clinical practice. Exercise was not further explored as prospectively collected data would better serve that analysis. Maternal dietary intake during gestation was estimated using a food frequency questionnaire which presents inherent limitations including misreporting and recall bias given its retrospective nature. Moreover, it would have been of added value if neonatal bone outcomes of interest were measured at birth. However, this was not feasible due to many family factors, including cultural views on research in newborns [60], fear, especially in first-time parents, as well as difficulties in managing to attend to a study visit and caring for the other children at once [61].

## 6. Conclusions

Our findings suggest that GWG is a modest determinant of neonatal bone mass, even when interpreted relative to body size. Additionally, although maternal 25(OH)D predicts neonatal 25(OH)D, it is not related to neonatal bone mass of healthy term AGA infants born to predominantly vitamin D sufficient mothers in our study. Nonetheless, given reliance in the extra-uterine environment on vitamin D stores acquired through maternal–fetal transfer, a longer-term follow-up is required to elucidate the impact of insufficient maternal vitamin D status on an offspring’s bone outcomes.

## Figures and Tables

**Table 1:** Maternal and infant characteristics according to maternal 25(OH)D and gestational weight gain categories.

	Maternal 25(OH)D (nmol/L)			Gestational Weight Gain			
	<50 (*n* = 45)	≥50 (*n* = 97)	*p*-value	Inadequate (*n* = 36)	Adequate (*n* = 46)	Excessive (*n* = 60)	*p*-Value
**Infant**							
							
Sex, *n* (%)			0.80				0.71
Male	27 (19.0)	56 (39.4)		21 (14.8)	29 (20.4)	33 (23.2)	
Female	18 (12.7)	41 (28.9)		15 (10.6)	17 (12.0)	27 (19.0)	
WAZ at birth	0.3 ± 0.8	0.1 ± 0.7	0.12	−0.3 ± 0.7 ^a^	0.2 ± 0.8 ^b^	0.4 ± 0.7 ^b^	0.0002
Age (month) at follow-up	0.7 ± 0.2	0.7 ± 0.2	0.31	0.7 ± 0.3	0.7 ± 0.2	0.7 ± 0.2	0.83
WAZ at follow-up	0.1 ± 0.8	−0.2 ± 0.8	0.13	−0.4 ± 0.8 ^a^	−0.0 ± 0.8 ^ab^	0.1 ± 0.7 ^b^	0.02
LAZ at follow-up	0.2 ± 1.0	−0.1 ± 0.9	0.05	−0.4 ± 0.9 ^a^	−0.0 ± 0.9 ^ab^	0.2 ± 0.9 ^b^	0.02
**Mother**							
Age at delivery (year)	31.8 ± 5.3	32.4 ± 4.0	0.45	31.9 ± 5.0	33.0 ± 3.8	31.7 ± 4.5	0.32
Gravida, *n* (%)			0.11				0.34
1	18 (12.7)	26 (18.3)		14 (9.9)	11 (7.8)	19 (13.4)	
>1	27 (19.0)	71 (50.0)		22 (15.5)	35 (24.6)	41 (28.8)	
Self-reported population group, *n* (%)			0.0003				0.12
White	15 (10.6)	64 (45.1)		15 (10.6)	26 (18.3)	38 (26.8)	
All other^†^	30 (21.1)	33 (23.2)		21 (14.8)	20 (14.1)	22 (15.4)	
Education, *n* (%)			0.0266				0.97
University	31 (21.8)	68 (47.9)		24 (16.9)	33 (23.2)	42 (29.6)	
College/Vocational	6 (4.2)	24 (16.9)		9 (6.3)	9 (6.3)	12 (8.5)	
Elementary/High school	8 (5.6)	5 (3.6)		3 (2.1)	4 (2.8)	6 (4.3)	
Family income, *n* (%)			0.15				0.37
≥ CAD 70,000	20 (14.1)	60 (42.3)		20 (14.1)	26 (18.3)	34 (23.9)	
< CAD 70,000	17 (12.0)	24 (16.9)		9 (6.3)	11 (7.8)	21 (14.8)	
Not reported	8 (5.6)	13 (9.1)		7 (4.9)	9 (6.3)	5 (3.6)	
Pre-pregnancy BMI			0.05				0.03
< 25 kg/m^2^	25 (17.6)	70 (49.3)		29 (20.4)	33 (23.2)	33 (23.2)	
≥ 25 kg/m^2^	20 (14.1)	27 (19.0)		7 (5.0)	13 (9.2)	27 (19.0)	

Data are reported as mean ± SD or *n* (%) and were analyzed using a mixed linear model for continuous variables accounting for the fixed effect studied and using Chi square or Fisher’s test for categorical variables. Distinct letter superscripts (^a,b^) indicate statistically significant differences between gestational weight gain categories (*p* < 0.05, post hoc adjustment); values that share a common superscript are not different from one another. ^†^ All other self-reported population groups include South Asian, Chinese, Black, Filipino, Latin American, Arab, Southeast Asian, West Asian, Korean, Japanese, and other. Abbreviations: 25(OH)D: 25-hydroxyvitamin D, WAZ: weight-for-age z-scores, LAZ: length-for-age z-scores, CAD: Canadian dollars.

## Data Availability

The data described in the manuscript will not be made available because permission to share data was not requested at the time of obtaining participant consent.

## Supplementary Materials

The following are available online at https://www.mdpi.com/article/10.3390/nu13124189/s1, Figure S1: Gestational weight gain relative to pre-gravid BMI expressed as continuous data and according to recommended categories., Table S1: Maternal nutrient intake from food and supplements during pregnancy according to gestational weight gain categories, Table S2: Correlates of neonatal bone mass tested using mixed linear model analysis, Table S3: Correlates of neonatal bone mass tested using mixed linear model analysis.
