# Supplementary material for: Maternal Vitamin D Status and Gestational Weight Gain as Correlates of Neonatal Bone Mass in Healthy Term Breastfed Young Infants from Montreal, Canada
Source: Nutrients. 2021 Nov 23;13(12):4189. doi: 10.3390/nu13124189 (PMC8708298; doi:10.3390/nu13124189)
Supplement: Supplementary file 1 [file nutrients-13-04189-s001.zip › nutrients-1453636-supplementary.pdf]

Supplementary material

Maternal Vitamin D Status and Gestational Weight Gain as Correlates of Neonatal Bone Mass in Healthy Term Breastfed Young Infants from Montreal, Canada

Nathalie Gharibeh [1], Maryam Razaghi [1], Catherine A. Vanstone [1], ShuQin Wei [2], Dayre McNally [3], Frank Rauch [4], Glenville Jones [5], Martin Kaufmann [5] and Hope A. Weiler [1,6]

[1] School of Human Nutrition, McGill University, Ste-Anne-de-Bellevue, QC H9X 3V9, Canada; nathalie.gharibeh@mail.mcgill.ca (N.G.); maryam.razaghi@mail.mcgill.ca (M.R.); catherine.vanstone@muhc.mcgill.ca (C.A.V.)
[2] Institut National de santé Publique du Québec, Montréal, QC G1V 5B3, Canada; shuqin.wei@inspq.qc.ca
[3] Department of Pediatrics, Children's Hospital of Eastern Ontario, University of Ottawa, Ottawa, ON K1H 8L1, Canada; dmcnally@cheo.on.ca
[4] Shriners Hospital for Children, Montréal, QC H4A 0A9, Canada; frauch@shriners.mcgill.ca
[5] Department of Biomedical and Molecular Sciences, Queen's University, Kingston, ON K7L 3N6, Canada; gj1@queensu.ca (G.J.); martin.kaufmann@queensu.ca (M.K.)
[6] Nutrition Research Division, Bureau of Nutritional Sciences, Health Products and Food Branch,
Health Canada, Ottawa, ON K1A 0K9, Canada

Table S1. Maternal nutrient intake from food and supplements during pregnancy according to gestational weight gain categories

	Gestational weight gain			
	**Inadequate (*n*=31/35)**	**Adequate (*n*=42/45)**	**Excessive (*n*=51/59)**	**p-value**
**Energy (kcal)**	2170 ±735	2161±952	2080±787	0.86
	1954 (1782-2426)	1964 (1508-2744)	1831 (1458-2466)	
**Protein (g)**	98±33	97±45	92±35	0.71
	87 (80-114)	90 (67-114)	84 (65-118)	
**Carbohydrates (g)**	292±134	280±141	270±105	0.74
	262 (228-310)	251 (178-336)	252 (186-338)	
**Fat (g)**	74±22	79±33	76±34	0.80
	70 (58-83)	72(54-92)	66 (50-96)	
**Vitamin D (IU)**	615 ± 252	646±285	613±229	0.80
	593 (503-766)	635 (468-809)	586 (479-788)	
**Calcium (mg)**	1359±426	1399±587	1364±503	0.93
	1233 (974-1673)	1289 (943-1695)	1287 (1034-1747)	
**Magnesium (mg)**	471±152	486±165	476±149	0.91
	431 (370-548)	444 (350-608)	459 (351-599)	
**Phosphorus (mg)**	1563±519	1571±710	1471±578	0.69
	1426 (1197-1898)	1388 (1040-1947)	1401(1003-1812)	

Data are reported as mean±SD and median (IQR). Differences between the means of the different GWG categories were analyzed using mixed linear model accounting for the fixed effect studied.

Distinct letter superscripts ([a,b,c]) indicate statistically significant differences between gestational weight gain categories ($p<0.05$, post hoc adjustment); values that share a common superscript are not different from one another.

Abbreviations: IU: international units.

Table S2. Correlates of neonatal bone mass tested using mixed linear model analysis

	Regression coefficients	*p*-values	95% CI
**Infant WB BMC g (R^2 0.43, R^2_{adj} 0.42)**			
**Infant Factors**			
Sex (male; referent: female)	5.70	0.0035	1.91, 9.48
Age (mo)	16.64	<0.0001	8.66, 24.62
LAZ	5.09	<0.0001	2.94, 7.24
**Maternal Factors**			
Serum 25(OH)D (<50 nmol/L; referent: ≥50 nmol/L)	2.96	0.19	-1.49, 7.40
GWG (insufficient; referent: adequate)	-5.14	0.0010	-10.22, -0.06
Pre-pregnancy BMI (<25 kg/m^2; referent: ≥25 kg/m^2)	2.43	0.25	-1.72, 6.59
Self-identified population group (all others; referent: white)	1.92	0.36	-2.36, 6.19
Education (elementary/high school; referent: university)	-1.61	0.13	-8.36, 5.13
Family yearly income (<70 000 CAD; referent: ≥70 000 CAD)	-1.91	0.0161	- 6.54, 2.72
**Infant WB BMC/weight g/kg (R^2 0.27, R^2_{adj} 0.26)**			
**Infant Factors**			
Sex (male; referent: female)	0.25	0.57	-0.61, 1.12
Age (mo)	-3.99	<0.0001	-5.81, -2.16
LAZ	-0.72	0.0044	-1.21, -0.23
**Maternal Factors**			
Serum 25(OH)D (<50 nmol/L; referent: ≥50 nmol/L)	0.82	0.11	-0.19, 1.84
GWG (insufficient; referent: adequate)	-0.77	0.0002	-1.93, 0.40
Pre-pregnancy BMI (<25 kg/m^2; referent: ≥25 kg/m^2)	0.50	0.29	-0.44, 1.45
Self-identified population group (all others; referent: white)	0.01	1.00	-0.97, 0.99
Education (elementary/high school; referent: university)	-0.70	0.0823	-2.25, 0.84
Family yearly income (<70 000 CAD; referent: ≥70 000 CAD)	-0.28	0.19	-1.34, 0.78
**Infant LS BMC g (R^2 0.28, R^2_{adj} 0.28)**			
**Infant Factors**			
Sex (male; referent: female)	-0.06	0.35	-0.18, 0.06
Age (mo)	-0.34	0.0104	-0.60, -0.08
LAZ	0.11	0.0024	0.04, 0.18

**Maternal Factors**			
Serum 25(OH)D (<50 nmol/L; referent: ≥50 nmol/L)	0.08	0.28	-0.07, 0.22
GWG (inadequate; referent: adequate)	-0.07	0.0051	-0.24, 0.09
Pre-pregnancy BMI (<25 kg/m^2; referent: ≥25 kg/m^2)	-0.05	0.43	-0.19, 0.08
Self-identified population group (all others; referent: white)	-0.02	0.79	-0.16, 0.12
Education (elementary/high school; referent: university)	-0.13	0.43	-0.35, 0.09
Family yearly income (<70 000 CAD; referent: ≥70 000 CAD)	-0.03	0.56	-0.19, 0.12
**Infant LS BMD g/cm^2 (R^2 0.29, R^2_{adj} 0.29)**			
**Infant Factors**			
Sex (male; referent: female)	-0.01	0.0236	-0.03, -0.00
Age (mo)	-0.06	<0.0001	-0.09, -0.04
LAZ	-0.00	0.74	-0.01, 0.01
**Maternal Factors**			
Serum 25(OH)D (< 50 nmol/L; referent: ≥50 nmol/L)	0.01	0.21	-0.01, 0.02
GWG (inadequate; referent: adequate)	-0.01	0.0018	-0.03, 0.01
Pre-pregnancy BMI (< 25 kg/m^2; referent: ≥ 25 kg/m^2)	-0.00	0.69	-0.02, 0.01
Self-identified population group as (all others; referent: white)	-0.01	0.28	-0.02, 0.01
Education (elementary/high school; referent: university)	-0.01	0.38	-0.04, 0.01
Family yearly income (<70 000 CAD; referent: ≥70 000 CAD)	0.00	0.82	-0.01, 0.02

Data are regression coefficients (95% CI) calculated using mixed linear model accounting for the fixed effects of infant's sex, age, and LAZ-score and mother's 25(OH)D categories, pre-pregnancy BMI categories, gestational weight gain categories, education, self-reported population group as well as family income.

Abbreviations: WB BMC: whole body bone mineral content, LAZ: length-for-age z-score, 25(OH)D: 25-hydroxyvitamin D, GWG: gestational weight gain, CAD: Canadian dollars, WB BMC/kg: whole body bone mineral content per kilogram body weight, LS BMC: lumbar spine bone mineral content, and LS BMD: lumbar spine bone mineral density.

Table S3. Correlates of neonatal bone mass tested using mixed linear model analysis

	Regression coefficients	*p*-values	95% CI
**Infant WB BMC g (R^2 0.42, R^2_{adj} 0.42)**			
**Infant Factors**			
Sex (male; referent: female)	5.57	0.0043	1.78, 9.36
Age (mo)	18.63	<0.0001	10.67, 26.58
LAZ	5.67	<0.0001	3.60, 7.74
**Maternal Factors**			
Serum 25(OH)D (per each nmol/L)	-0.04	0.32	-0.12, 0.04
GWG (per each kg)	0.64	0.0005	0.29, 0.99
Pre-pregnancy BMI (per each kg/m^2)	0.39	0.0778	-0.04, 0.82
Self-identified population group (all others; referent: white)	2.47	0.26	-1.83, 6.78
Education (elementary/high school; referent: university)	-1.41	0.16	-8.11, 5.30
Family yearly income (<70 000 CAD; referent: ≥70 000 CAD)	-2.34	0.0051	-6.99, 2.30
**Infant WB BMC/weight g/kg (R^2 0.26, R^2_{adj} 0.25)**			
**Infant Factors**			
Sex (male; referent: female)	0.20	0.65	-0.67, 1.07
Age (mo)	-3.51	0.0002	-5.33, -1.68
LAZ	-0.57	0.0174	-1.05, -0.10
**Maternal Factors**			
Serum 25(OH)D (per each nmol/L)	-0.01	0.20	-0.03, 0.01
GWG (per each kg)	0.16	0.0001	0.08, 0.24
Pre-pregnancy BMI (per each kg/m^2)	0.09	0.07	-0.01, 0.19
Self-identified population group (all others; referent: white)	0.14	0.78	-0.85, 1.13
Education (elementary/high school; referent: university)	-0.63	0.10	-2.17, 0.90
Family yearly income (<70 000 CAD; referent: ≥70 000 CAD)	-0.35	0.08	-1.41, 0.72
**Infant LS BMC g (R^2 0.28, R^2_{adj} 0.27)**			
**Infant Factors**			
Sex (male; referent: female)	-0.06	0.33	-0.18, 0.06
Age (mo)	-0.29	0.0289	-0.55, -0.03
LAZ	0.13	0.0002	0.06, 0.20

**Maternal Factors**			
Serum 25(OH)D (per each nmol/L)	-0.0002	0.85	-0.003, 0.002
GWG (per each kg)	0.02	0.0031	0.01, 0.02
Pre-pregnancy BMI (per each kg/m^2)	0.02	0.0045	0.01, 0.03
Self-identified population group (all others; referent: white)	0.01	0.85	-0.13, 0.15
Education (elementary/high school; referent: university)	-0.13	0.43	-0.34, 0.09
Family yearly income (<70 000 CAD; referent: ≥70 000 CAD)	-0.04	0.34	-0.19, 0.12
**Infant LS BMD g/cm^2 (R^2 0.30, R^2_{adj} 0.29)**			
**Infant Factors**			
Sex (male; referent: female)	-0.02	0.0201	-0.03, 0.00
Age (mo)	-0.06	<0.0001	-0.08, -0.03
LAZ	-0.001	0.83	-0.006, 0.008
**Maternal Factors**			
Serum 25(OH)D (per each nmol/L)	0.00004	0.75	-0.0003, 0.0002
GWG (per each kg)	0.002	0.0005	0.001, 0.003
Pre-pregnancy BMI (per each kg/m^2)	0.002	0.0034	0.001, 0.002
Self-identified population group as (all others; referent: white)	-0.004	0.58	-0.019, 0.010
Education (elementary/high school; referent: university)	-0.01	0.38	-0.04, 0.01
Family yearly income (<70 000 CAD; referent: ≥70 000 CAD)	0.003	0.63	-0.013, 0.019

Data are regression coefficients (95% CI) calculated using mixed linear model accounting for the fixed effects of infant's sex, age, and LAZ-score and mother's 25(OH)D concentration, pre-pregnancy BMI, gestational weight gain, education, self-reported population group as well as family income.

Abbreviations: WB BMC: whole body bone mineral content, LAZ: length-for-age z-score, 25(OH)D: 25-hydroxyvitamin D, GWG: gestational weight gain, CAD: Canadian dollars, WB BMC/kg: whole body bone mineral content per kilogram body weight, LS BMC: lumbar spine bone mineral content, and LS BMD: lumbar spine bone mineral density.

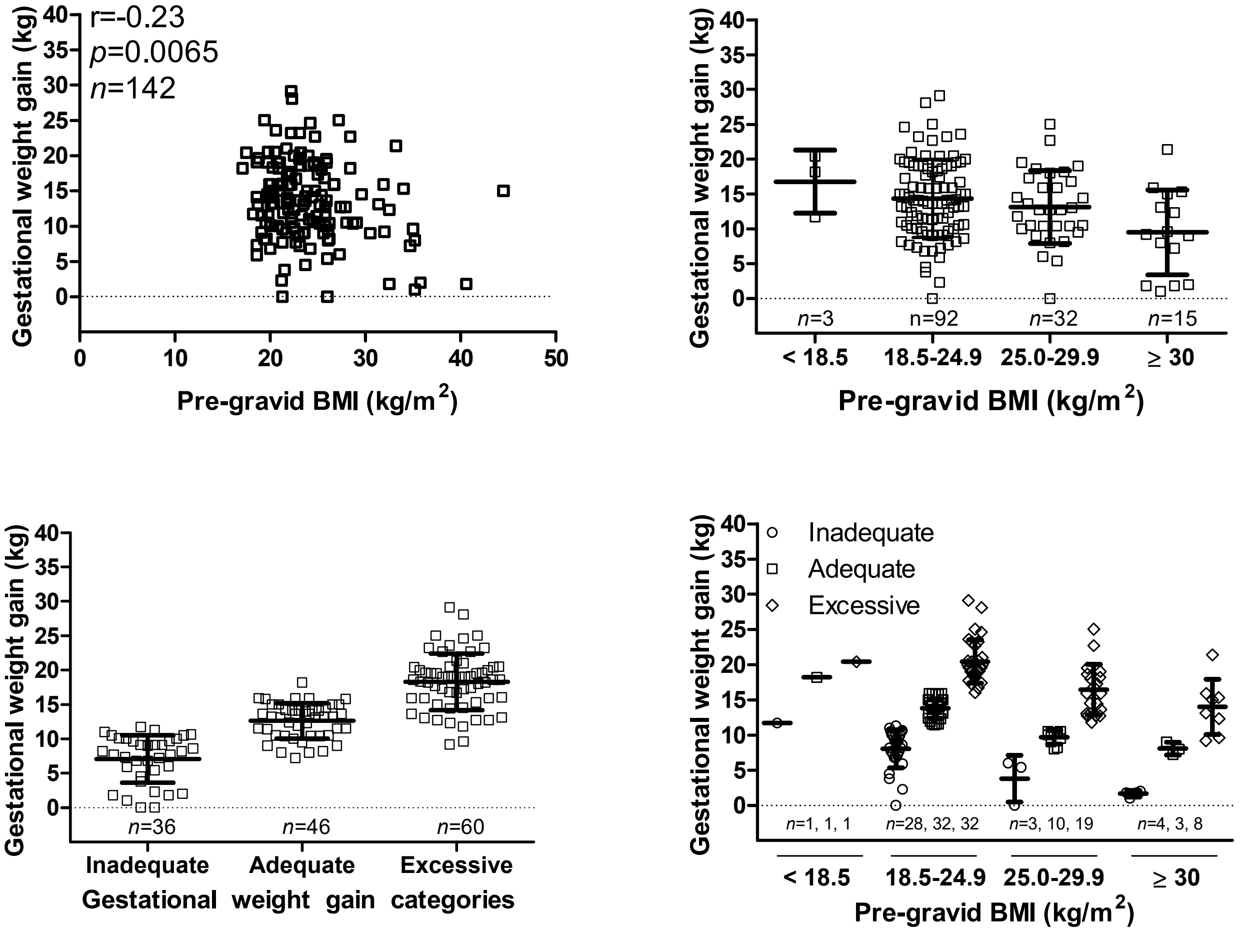

Figure S1. Gestational weight gain relative to pre-gravid BMI expressed as continuous data and according to recommended categories. Data are reported as mean ± SD. Data were analyzed using Pearson correlation for gestational weight gain (kg) according to pre-gravid BMI (kg/m^2). Pregnancy weight gain (kg) is descriptively shown relative to pre-gravid BMI categories and expressed as inadequate, adequate or excessive according to pre-gravid BMI categories.
